# Intestinal parasite infections and associated factors among inmates of Arba Minch prison, southern Ethiopia: cross sectional study

**DOI:** 10.1186/s12879-019-4703-y

**Published:** 2019-12-30

**Authors:** Gemechu Ameya, Zerihun Zerdo, Mihret Tesfaye, Chimdo Jabesa, Abayneh Awaje, Kaleb Dejene, Petros Shika, Mohammed Eshetu

**Affiliations:** 1 Department of medical laboratory sciences, College of medicine and health sciences, Kotebe Metropolitan University, P.O. Box: 31248, Addis Ababa, Ethiopia; 2 Department of medical laboratory sciences, College of medicine and health sciences, Arba Minch University, Arba Minch, Ethiopia; 3 Department of Medical laboratory Science, Hiwot Fana specialized referral hospital, Haramaya University, Harar, Ethiopia; 4 Department of Medical laboratory Science, Shone Primary Hospital, Shone, Ethiopia; 5 Department of Medical laboratory Science, Sawla General Hospital, Sawla, Ethiopia; 6 Department of Medical laboratory Science, Chire Primary Hospital, Chire Kanama, Ethiopia; 7 Department of Medical laboratory Science, Semera Health Center, Semera, Afar Ethiopia

**Keywords:** Intestinal parasite, Multiple parasitic infection, Prisoners, Protozoa, Soil transmitted helminths

## Abstract

**Background:**

Intestinal parasitic infection is one of the parasitic infections affecting people living in prison. Helminths and intestinal protozoan infections are the most common parasitic infection that may cause serious life-threatening diseases in inmates living in developing countries. This study was aimed to investigate the prevalence and associated factors of intestinal parasitic infections (IPIs) among inmates living in Arba Minch prison, southern Ethiopia.

**Methods:**

Institutional based cross sectional study was conducted on Arba Minch inmates, southern Ethiopian. Pre-tested semi-structured questionnaire was used to gather the data of socio-demographic characteristics, hygiene status of the prisoners, sanitation condition of the prison, and associated factors for IPIs by face to face interview. Direct wet-mount examination and formol-ether sedimentation techniques were used to examine intestinal parasitic infection from stool specimens. Binary logistic regression analysis was used to see the association between different variables and the IPI. Odds ratio with 95% CI was computed to determine the presence association and strength of the associated factors.

**Result:**

A total of 320 prisoners were participated in this study. Of these, 154(48.1%) of them were infected with one or more intestinal parasites. Eight different intestinal parasites species were identified and *Giardia lamblia* was the predominant parasite. Among infected inmates, nearly one out of four of them had multiple parasitic infections dominated by *Giardia lamblia* and *E. histolytica/dispar* co-infection. Sleeping in group [AOR = 1.9; 95% CI: (1.0–3.8)], married prisoners [AOR = 1.8; 95% CI: (1.1–2.9)], and hand washing habits after handling soil [AOR = 2.4; 95% CI: (1.0–5.6)] were independently associated with IPI.

**Conclusion:**

High prevalence of intestinal parasitic infection was detected in Arba Minch inmates, southern Ethiopian. Absence of hand washing, marital status, and way of sleeping were the factors associated with the IPI. Implementation of mass drug administration, education on water, sanitation and hygiene (WASH) and periodic screening of intestinal parasitic infection is very important to reduce the high prevalence IPIs in prison.

## Background

Intestinal parasite infection (IPI) particularly schistosomiasis and soil transmitted helminthiasis are the major neglected tropical diseases causing significant morbidity in most countries. Although different efforts were undertaken to minimize the burdens of the disease, they are causing significant problem in developing country. IPIs remains one of the greatest health problems estimated to be over a billion of people infected by at list one of the species [1, 2]. Geo-helminths and enteric protozoa are readily passed from person to person via the fecal-oral route in various situations and present throughout the world in varying degrees of prevalence. Over 70 species of protozoan and helminth parasites can infect humans via contaminated food and water [3]. Among intestinal parasitic infection, schistosomiasis and soil transmitted helminthiasis are more prevalent throughout tropical countries [4, 5].

More than 24% of the world population is infected by soil-transmitted helminthes, and about 50% of people in developing countries are believed to be infected by helminthes [6]. In industrialized countries protozoan infections are associated with the use of common pools in water parks and handling of infected pets. However, in developing countries the infection is mainly associated with low level of environmental and personal hygiene, contamination of food and drinking as well as poor toilet facility [7].

Helminthic infections are the second most predominant causes of outpatient morbidity in Ethiopia. Ethiopian federal ministry of health report indicated that the prevalence of parasitic infections were high in low altitudes [8]. Prevalence of intestinal parasite infection in population living in highland and lowland of Gamo area was 27.7% [9]. Other studies conducted in different part of Ethiopia also showed a varied prevalence that ranged from 29.1 to 49.4 [10–13]. The problem is more sever for inmates due to different reasons. The inmates’ right is restricted, their living style favor intestinal parasitic infection and they may not have the access of health care timely. Inmates in developing countries are neglected and they are not benefited from health program given for other citizen [14].

The current mass drug administration strategy show a significant improvement in the burden of intestinal helminthes in school aged children [15]. However, the prevalence of the intestinal parasite in non-targeted population remains high. People living in developing countries’ prisons are not benefited from the health care system given for other population. Because of inmates’ activities are limited, they may not get on time health care and treatment [16]. More than 10 million people are incarcerated throughout the world with the majority lives in developing country. With this high number, they could be the source of infection for other people through their visitors. According to the 2012 world prison report, Ethiopia was leading in prisoner number in East Africa [17]. The varied composition of inmates who came from different regions of Ethiopia could create a fertile ground for transmission of different species of intestinal parasites.

Studies conducted in different prisons showed a varied prevalence of intestinal parasitic infection. In Malaysia prison the prevalence of intestinal parasite was about 26.5% [18], in Brazil 20.2% [19], and in Kenya it was 24.7% [20]. Few studies were conducted in Ethiopia and the prevalence of intestinal parasitic infection was relatively high compared to other studies. The estimated prevalence of intestinal parasitic infection in Shewa Robit prison was 61.8% [21] while the study in Bedele prison indicated 64% prevalence [22]. However, there was no study conducted in southern part of Ethiopia.

Inmates in developing countries are more likely vulnerable to intestinal parasite infection diseases due to a number of factors. Albeit the factors varied from place to place, the most common are poor health care, overcrowding, high level of stress and inadequate or poor nutritional quality, and low living standard as compared to the general population [18–22]. Although helminths infection is very common in Ethiopia, study related to prevalence and associated factors of the infection in inmate are not well investigated in the study area. This study was aimed to assess the prevalence of intestinal parasite infection and associated factors of the infection among Arba Minch prison inmates, Southern Ethiopia.

## Methods

### Study design, settings and period

Institutional based cross sectional study was conducted on randomly selected prisoners of Arba Minch town. This study was conducted in prison located in Arba Minch city. Arba Minch is a administrative seat of Gamo zone, Southern Nations, Nationalities, and Peoples’ Regional state. The prison is located in Sikella sub-city and the total number of prisoners was 2323 during the data collection period. The majority of the inmates were male. The study was conducted from March to June, 2018.

### Study population

The study subjects were recruited from prisoners resident in Arba Minch prison. Inmates who had taken anti-parasitic medications in the last 2 weeks of data collection period, a prisoner unable to respond to questions in the questionnaire, and those who were incarcerated within the last 1 month of data collection period were excluded from the study.

### Sample size determination and sampling technique

The sample size for the present study was determined by using single population proportion formula based on the study conducted in Shewa Robit prison in which the prevalence of IPIs was 72.7% (*p* = 0.73) [21]. The 95% of level of confidence and 5% precision was used. Then by considering 15% for non-response rate the total sample size became 351. Then study subjects were recruited in the study by using systematic random sampling technique. The male and female participants were proportionally allocated in the study based on their total number in the prison.

### Study variables

The outcome variable of study was intestinal parasitic infection. The independent variables such as socio-demographic and socio-economic characteristics, sanitation and hygiene status of participants and other potential associated factors were included.

### Data collection

A pretested semi-structured questionnaire administered through face to face interview was used to collect data on socio demographic characteristics and associated factors for intestinal parasite infection (see Additional file 1). During data collection, each prisoner was asked separately and the confidentiality of the information was well explained to minimize social desirability bias. To minimize contamination of information, data of all participants found in one room were collect in the same period.

### Specimen collection and examination

About 5gm of stool specimens was collected from study participants in pre-labeled, leak-proof plastic stool cups. The fecal specimens were physically examined and the consistency (watery diarrheic, diarrheic bloody and formed) of the samples was recorded and used for input in microscopic examination. Wet-mount preparation was prepared immediately after collection in prisoner clinic laboratory using normal saline and microscopic examination was made to identify parasite ova, motile trophozoites of intestinal protozoa and larvae of *S. stercoralis*.

To enhance sensitivity of the microscopic examination, stool concentration was performed using 10% formalin. Formal ether sedimentation technique was carried out by mixing stool specimen about the size of a walnut with 10 mL of normal saline and the suspension was centrifuged at 2,500 rpm for 5 minutes. Then the supernatant was discarded and sediment was washed, and again centrifuged at 2,500 rpm for 5 minutes. After 7 mL of 10% formalin was added and kept for 5 minutes, 2 mL of ethyl acetate was added. Then the suspension was centrifuged at 1500 rpm for 10 minutes. After carefully decanting the top three layers, the remaining sediment was mixed and pipetted using pasture pipette. Finally wet-mount from the suspension was prepared on slide for microscopic examination.

### Data processing and analysis

The data collected from the study area were entered in excel sheet before being exported to SPSS version 22. Analyses of the variables were made using descriptive statistics and bivariate logistic regression analysis. Bivariate logistic regression analysis was employed to determine the association between various variables and intestinal parasite infection. Univariable logistic regression analysis was conducted and a variable with *p*-value ≤0.20 were included in multivariable logistic regression analysis to identify variables that independently associated with the intestinal parasite infection. Adjusted odds Ratios (AOR) and their 95% CI were used to look into the presence and strength of association between the dependent and independent variables.

## Results

### Socio-demographic characteristics of participants

A total of 320 inmates were participated in the study with 91.2% response rate. Age of the participants ranged from 15 to 82-year-old with the mean age of 31.98 ± 13.48 years. About two-thirds of participants belong to the age group of 30-year-old and younger and majority of them were male. Nearly two-third of the participants were came from rural area and most of them were lived on agricultural work before incarcerated. A quarter of study participants were illiterate while large number of them were unemployed before imprisoned. The number of prisoners per room was ranged from 15 to 250 with average of 142 prisoners per a room (Table 1).

**Table 1:** Socio-demographic characteristics of study participants (*n* = 320)

Variables	Categories	No.	%
Sex	Male	305	95.3
	Female	15	4.7
Age	≤30	200	62.5
	31–50	76	23.8
	> 50	44	13.8
Marital status	Married	212	66.3
	Single	108	33.8
Original residence	Urban	123	38.5
	Rural	197	61.5
Educational status of prisoners	Illiterate	83	25.9
	Primary	156	48.8
	Secondary	64	20.0
	College & above	17	5.3
Occupation before imprisoned	Unemployed	116	36.3
	Farmer	169	52.8
	Gov’t Emp	14	4.4
	Private work	21	6.6
Duration of incarceration	< 2 years	205	64.1
	> 2 years	115	35.9
Current income	No income	137	42.8
	< 500	106	33.1
	501–1000	61	19.1
	> 1000	16	5.0

### Sanitation and hygienic practice of the inmates

Majority of the participants had hand washing habit after visiting toilet but only half of them had habit of washing with soap. About one-fifth of the prisoners had habit of eating fruit without washing and meat before cooking. Nearly one-third of study participants had untrimmed finger nail. Drinking water source was different for male and female participants; the male participants had drunk water stored in tanker while the female participants had drunk from running pipe water. About 10% of the inmates’ rooms had no waste disposal container (Table 2).

**Table 2:** Sanitation, hygeine practice and medical condition of study participants (*n* = 320)

Variables	Categories	No.	%
Having hand practice after toilet	Yes	300	93.8
	No	20	6.3
Frequency hand washing after toilet	Always	121	37.8
	Most of time	77	24.1
	Some times	94	29.4
	Rarely	11	3.4
Using soap for washing hand	Yes	158	52.7
	No	142	47.3
Hand washing practice after handling soil	Yes	293	91.6
	No	27	8.4
Eat vegetables before washing	Yes	262	81.9
	No	58	18.1
Eat raw or undercooked meat	Yes	52	16.3
	No	268	83.8
Finger nail of participant	Trimmed	215	67.2
	Not trimmed	105	32.8
Frequency of shoe wearing	Always	153	7.8
	Most of time	57	17.8
	Some times	105	32.8
	Rarely	5	1.6
Source of drinking water	Tanker	305	95.3
	Pipe	15	4.7
Waste disposal container in the inmates room	Yes	289	90.3
	No	31	9.7
Open defecation practice	Yes	6	1.9
	No	314	98.1
Having of chronic diseases	Yes	54	16.9
	No	266	83.1
Type of chronic diseases (*n* = 54)	HIV	10	3.1
	DM	6	1.9
	CVI	25	7.8
	Hypertension	11	3.4

### Intestinal parasitic infections among inmates

**Fig. 1:**
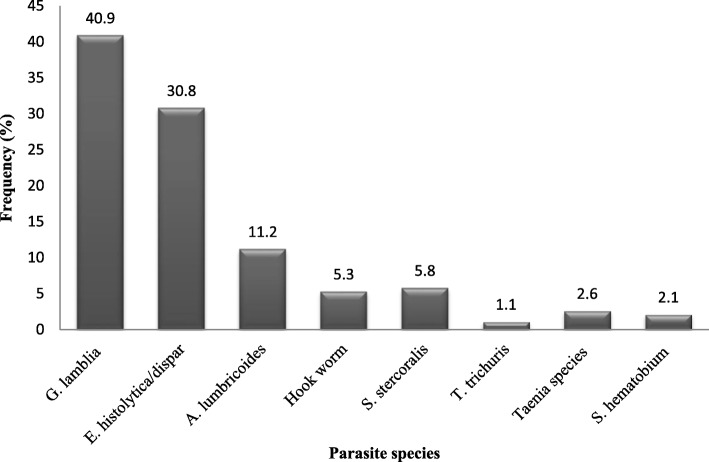
Intestinal parasite species identified in Arba Minch prisoners, southern Ethiopian

The overall prevalence of intestinal parasite among Arba Minch prison inmates was 48.1% [95% CI: (42.6–53.6)]. Eight different intestinal parasite species were identified in study participants (Fig. 1). *Giardia lamblia* was the predominant (41.3%) intestinal parasite followed by *E. histolytica* which accounts about 30.3% of the identified intestinal parasites. *A. lumbricoides* and Hookworms were the predominant intestinal helminths with 17 and 6.9% prevalence, respectively. About 23% of prisoners were infected with more than one parasite. The majority of the co-infection was by *G. lamblia* and *E. histolytica* followed by *A. lumbricoides* and *E. histolytica* (Table 3).

**Table 3:** Frequency of co-infection of intestinal parasite in the study participants (*n* = 320)

Co-infection	Frequency	Percent
*G. lamblia* and *E. histolytica*	18	51.4
*E. histolytica* and *A. lumbricoides*	9	25.7
*G. lamblia* and *S. hematobium*	4	11.4
Taenia species and *G. lamblia*	3	8.6
Hook worm, *T. trichuris,* and *S. stercoralis*	1	2.9
Total	35	100

### Factors associated with intestinal parasitic infection of the inmates

As mention in the method part logistic regression analysis was used to find out the associated factors. In univariable analysis resident of the inmate before incarceration, marital status, number of inmates per incarceration room, availability of waste disposal container in incarceration room, hand washing habit after handling soil, having chronic disease, and way of sleeping were transferred into multivariable logistic regression together based on criteria stated in method section.

In multivariable logistic regression, hand washing practice after handling soil, marital status and prisoners’ way of sleeping were independently associated with intestinal parasite infection of the inmates. Married inmate had two times odds of intestinal parasitic infection than single prisoners [AOR = 1.8; 95% CI: (1.1, 2.9)]. Inmates who had no hand washing practice after handling soil had about two time odds of infection with intestinal parasite [AOR = 2.4; 95% CI: (1.0, 5.6)]. The prisoners who were sleeping in group had about two times more chance of intestinal parasitic infection than those who sleep individually [AOR = 1.9; 95% CI: (1.0, 3.8)] **(**Table 4**)**.

**Table 4:** Factors associated with intestinal parasite infections of the inmates

Variable	IP infected	None IP infected	COR (95% CI)	AOR (95% CI)	*P*-Value
Previous residence					
Urban	52	71	Ref.		
Rural	102	95	1.5 (0.9–2.3)		
Marital status					
Married	112	100	1.8 (1.1–2.8)	1.8 (1.1–2.9)	0.014*
Single	42	66	Ref.	Ref.	
Availability of Waste disposal container					
Available	143	146	Ref.		
Not available	11	20	0.6 (0.3–1.2)		
Way of sleeping					
Individual	17	30	Ref.	Ref.	
In group	137	136	1.8 (0.9–3.4)	1.9 (1.0–3.8)	0.039*
Number of inmates per room					
< 100	16	23	Ref.		
100–150	73	82	1.3 (0.6–2.6)		
> 150	65	61	1.5 (0.7–3.2)		
Hand washing practice after handling soil					
Yes	145	148	Ref.	Ref.	
No	9	18	0.5 (0.2–1.2)	2.4 (1.0–5.6)	0.047*
Having chronic diseases					
Yes	21	33	0.6 (0.4–1.2)	0.6 (0.3–1.1)	0.091
No	133	133	Ref.	Ref.	

*: Independently associated with intestinal parasite infection; *AOR* Adjusted Odd Ratio, *COR* Crude Odd Ratio, *Ref*. Reference

## Discussion

Prison conditions should promote the health of both prisoners and prison staff and providing proper health care for inmates is also very important. The issue of IPI in vulnerable population particularly inmate seeks special attention. The prevalence of intestinal parasitic infection among inmates in Arba Minch prison, southern Ethiopia was 48.1% [95% CI: (42.6–53.6)]. The current finding was slightly lower than study conducted in Showa Robit [21] and Bedele prisons [22]. The observed difference may be due to difference in the activities of the inmates. In the Showa Robit study, participants involved in tobacco farmer were included and this increases the chance of frequently contracting with soil. In addition, unlike our finding the frequency of soil transmitted helminths were high in the Showa Robit study. On the other hand, our find is higher than the other studies conducted in Malaysia [18], Brazil [19], and Kenya prisoners [20]. The high prevalence obtained in our study may be due to the lack of sanitation in the compound, lack of clean water for inmates, and high number of prisoner per room.

In this study 8 different intestinal parasite species were identified. The predominant intestinal parasite was protozoa particularly *G. lamblia* followed by *E. histolytica/dispar.* Similarly, in the study conducted in Brazil prison *G. lamblia* and *E. histolytica/dispar* were the most frequent intestinal parasite [19]. Of infected inmates, about one quarter of them had more than one intestinal parasite infection. *Giardia lamblia* and *E. histolytica* co-infection was the most frequent in current study. Rop et al study showed that there was low number of mixed infection (3.5%) in Kisii prison of Kenya as compared to our finding [20]. This high protozoa co-infection in our study may be due to unimproved drinking water in the prison. Ineffective treatment and exchange in the life cycle of protozoa between cyst and trophozoite could be the other possible reason for the high protozoa infection. In contrary, the study conducted in Malaysia prison reported low prevalence of protozoa infection [18]. In the study conducted in Shewa Robit prison, *E. histolytica/dispar* was the predominant intestinal parasite and the frequent co-infection observed was *E. histolytica/dispar* and *A. lumbricoide* [21]. The observed difference may be due to the difference in study population; in Shewa Robit study, the participants were sourced from prison and tobacco farmer.

In view of the fact that intestinal parasite infection has faeco-oral route of transmission, effective hand washing is very important to prevent the infection. In our study, absence of hand washing after handling soil is significantly associated with intestinal parasitic infection. In this study majority of the participants were came from rural area and they had less awareness on the route of transmission. Other studies also revealed as ineffective hand washing are risk for intestinal parasitic infection. A factorial cluster randomized controlled trial study showed that hand washing with soap has significant impact in reducing the infection of intestinal parasite [23]. Similar to current study, study conducted in Kenya prison also showed association between hand washing and intestinal parasitic infection [20]. In addition, the low access to improved water supply, poor medication, low economic background and poor hygiene in the person favor this association.

The marital status of the prisoner was determinant factor for IP infection of the study participants. Married inmate had two times odds of IP infection than single prisoners. Most probably married individuals have large family than singles. Studies also showed that large family has more chance intestinal parasite infection than that of small family [23, 24]. Similar studies conducted in Accra and Bahir Dar showed that intestinal parasitic infection was associated with the family size [24, 25]. In addition, majority of the participants those categorized under married status were also came from rural area.

In the current study, prisoners’ way of sleeping was another factor that is associated with intestinal parasite infection in the prison. Those prisoners who sleep in group had two times more chance of intestinal parasitic infection than inmates who sleep separately. Crowded life style is a risk for intestinal parasitic infection [26]. Sleeping on ground may facilitate transmission of soil transmitted parasites. In addition, this may be due to the majority of study participants who sleep in group were prisoners incarcerated for the first 2 years where infection before incarceration might have contributed to the high prevalence in this group of prisoners.

This study has some of limitations. One of the limitations was lack of antigen test to differentiate *Entamoeba histolytica* and *Entamoeba dispar* cyst. Limitation related to social desirability bias, and recall bias can be expected in this study. However, to minimize these biases the prisoners were asked individually and the purpose of the study was well explained before the data collection.

## Conclusion

The prevalence of intestinal parasitic infection in Arba Minch prison is comparable with other inmates’ IP infection in developing country. Protozoan infection was higher among identified parasitic species. Marital status of the inmate, hand washing habit after handling soil and sleeping in group were independently associated with intestinal parasitic infection of the prisoners. The effective communication between prison governing body and the prison clinics is required to decrease the prevalence of the infection among inmates. Mass drug administration, giving periodic WASH education in prison and modifying way of sleeping for inmates are mandatory to reduce the observed high prevalence of intestinal parasitic infection.

## Supplementary information

**Additional file 1.** Questionnaire used during collecting the data.

## Data Availability

The datasets supporting the conclusions of this article are included within the article.

## Abbreviations

AOR: Adjusted odd ratio
CI: Confidence interval
COR: Crude odd ratio
IP: Intestinal parasitic
IPI: Intestinal parasitic infection
SPSS: Statistical package for social sciences
STH: Soil transmitted helminthes
WASH: Water, sanitation and hygiene

## References

[1] World Health Organization. Intestinal worms. Accessed 1 Oct 2018. Available on: http://www.who.int/intestinal_worms/more/en/.

[2] World Health Organization (2012). Research priorities for helminth infections. World Health Organ Tech Rep Ser.

[3] Mandal FB (2015). Human parasitology, second edition.

[4] World Health Organization (2002). Prevention and control of schistosomiasis and soil transmitted helminthiasis.

[5] Heelan JS (2004). Cases in human parasitology.

[6] World Health Organization. Soil-transmitted helminth infections. Accessed 15 Oct 2018. Available on: http://www.who.int/news-room/fact-sheets/detail/soil-transmitted-helminth-infections

[7] Federal Democratic Republic of Ethiopia Ministry of Health (2005). National hygiene and sanitation strategy to enable 100% adoption of improved hygiene and sanitation.

[8] FDRE Ministry of Health (2010). Comprehensive health service directory.

[9] Wegayehu T, Tsalla T, Seifu B, Teklu T (2013). Prevalence of intestinal parasitic infections among highland and lowland dwellers in Gamo area, South Ethiopia. BMC Public Health.

[10] Andargie G (2008). Prevalence of bacteria and intestinal parasites among food-handlers in Gondar town, Northwest Ethiopia. J Health Popul Nutr.

[11] Nigusse D, Kumie A (2012). Food hygiene practices and prevalence of intestinal parasites among food handlers working in Mekelle university student’s cafeteria, Mekelle. Global Adv Res J Soc Sci.

[12] Tamirat T, Mebrie G (2014). Prevalence and predictors of intestinal parasites among food handlers in Yebu town, Southwest Ethiopia. PLoS One.

[13] Feleke DG, Wage EK, Getachew T, Gedefie A (2019). Intestinal parasitic infections and associated factors among street dwellers’ in Dessie town, North-East Ethiopia: a cross sectional study. BMC Res Notes.

[14] World health organization. Prison and Health. Enggist S, Møller L, Galea G, Udesen C. WHO Regional Office for Europe, 2014. Available on: http://www.euro.who.int/__data/assets/pdf_file/0005/249188/Prisons-and-Health.pdf. Accessed 22 Feb 2019.

[15] Webster JP, Molyneux DH, Hotez PJ, Fenwick A (2014). The contribution of mass drug administration to global health: past, present and future. Phil Trans R Soc B.

[16] Weldeyohannes BT (2017). Reforming prison policy to improve women specific health and sanitary care conditions of prisons in Ethiopia. Wm Mary J Women L.

[17] Walmsley R (2015). World Prison Population List.

[18] Angal L, Mahmud R, Samin S, Yap N, Ngui R, Amir A (2015). Determining intestinal parasitic infections (IPIs) in inmates from Kajang prison, Selangor, Malaysia for improved prison management. BMC Infect Dis.

[19] Curval LG, França AO, Fernandes HJ, Mendes RP, de Carvalho LR, Higa MG (2017). Prevalence of intestinal parasites among inmates in Midwest Brazil. PLoS One.

[20] Rop DC, Nyanchongi BO, Nyangeri J, Orucho VO (2016). Risk factors associated with intestinal parasitic infections among inmates of Kisii prison Kenya, Ropet al. BMC Res Notes.

[21] Mamo H (2014). Intestinal parasitic infections among prison inmates and tobacco farm workers in Shewa Robit, North-Central Ethiopia. PLoS One.

[22] Terefe B, Zemene E, Mohammed AE (2015). Intestinal helminth infections among inmates in Bedele prison with emphasis on soil-transmitted helminthes. BMC Res Notes.

[23] Forson AO, Arthur I, Ayeh-Kumi PF (2018). The role of family size, employment and education of parents in the prevalence of intestinal parasitic infections in school children in Accra. PLoS One.

[24] Hailegebrial T (2018). Undernutrition, intestinal parasitic infection and associated risk factors among selected primary school children in Bahir Dar, Ethiopia. BMC Infect Dis.

[25] Phiri K, Whitty CJ, Graham SM, Ssembatya-Lule G. Urban/rural distance in prevalence of intestinal helminths in southern Malawi. Ann Top Med Parasite. 2000;94:381–7. 10.1080/00034983.2000.11813553 10945048

[26] Mahmud MA, Spigt M, Bezabih AM, Pavon IL, Dinant G-J, Velasco RB (2015). Efficacy of hand washing with soap and nail clipping on intestinal parasitic infections in school-aged children: A factorial cluster randomized controlled trial. PLoS Med.

