# Supplementary material for: Intestinal parasite infections and associated factors among inmates of Arba Minch prison, southern Ethiopia: cross sectional study
Source: BMC Infect Dis. 2019 Dec 30;19:1086. doi: 10.1186/s12879-019-4703-y (PMC6937967; doi:10.1186/s12879-019-4703-y)
Supplement: Supplementary file 1 — Additional file 1. Questionnaire used during collecting the data. [file 12879_2019_4703_MOESM1_ESM.docx]

**Questionnaire**

**Part I: Question related to Socio-demographic information**

| **No** | **Characteristics** | **Choices** | **Remark** |
| --- | --- | --- | --- |
| 101 | Gender | 1. Male 2. Female |  |
| 102 | Age (In year) | _____________________ |  |
| 103 | Previous Residence of the prisoner | 1. Urban 2. Rural |  |
| 104 | Marital status | 1. Single 2. Married 3. Divorced 4. Widow |  |
| 105 | Educational status | 1. Illiterate 2. Primary education 3. Secondary education 4. College and above |  |
| 106 | Previous Occupation | 1. Farmer 2. Government employer 3. Unemployed 4. Merchant 5. House wife 6. Other |  |
| 107 | Current month income of prisoner (In Eth.  Birr) | 1. <500 2. 500-1000 3. 1000-1500 4. >1500 |  |
| 108 | Number of prisoner in your room | ____________ |  |
| 109 | Duration of  incardination (In year) | ___________________________ |  |

**Part II: Behavior factor of the prisoner and question related to IPIs associated factors**

| **No.** | **Characteristics** | **Choices** | **Remark** |
| --- | --- | --- | --- |
| 201 | Do you wash your hand after using toilet? | 1. Yes 2. No |  |
| 202 | Do you wash your hand after handling soil? | 1. Yes 2. No |  |
| 203 | How often you wash your hand in the above situation | 1. Always 2. Most of the time 3. Some times 4. Rarely |  |
| 204 | How do you wash your hand | 1. With soap and clean water 2. With soap and unclean water 3. Without soap and clean water 4. Without soap and unclean water |  |
| 205 | How you eat fruit | 1. After washing 2. Without washing |  |
| 206 | Meat eating habit | 1. Raw 2. Cooked |  |
| 207 | Finger nail trimming status | 1. trimmed 2. untrimmed | Observation |
| 208 | Habit of drug usage during abdominal discomfort | 1. using drug after Lab. Diagnosis 2. using drug without Lab. Diagnosis 3. using other alternative medicine   (traditional medicine) |  |
| 209 | How often you wearing shoe | 1. Always 2. Most of the time 3. Some times 4. Rarely |  |
| 210 | Source of drinking water | 1. from water pipe 2. from tanker 3. stored container 4. other |  |
| 211 | Is there waste disposal container in your room | 1. Yes 2. No |  |
| 212 | Is there habit of defecation other than toilet | 1. Yes 2. No | If No for this question skip the next question |
| 213 | If yes for the above question what kind of means is used | _________________ | Specify |
| 214 | Type of bedding | 1. On pad 2. On mattress 3. On flour |  |
| 215 | Style of sleeping | 1. Individually 2. By group |  |
| 216 | Type of bathroom | 1. Single 2. By group |  |
| 217 | Do you have chronic infection? | 1. Yes 2. No |  |
| 218 | If yes for no 217 question, what type of chronic infection do you have? | 1. HIV 2. DM 3. Cardio vascular infection 4. other | Specify |
